# MODELING TRAUMATIC BRAIN INJURY (TBI)–INDUCED NEURODEGENERATION USING HIPSC-CORTICAL ORGANOIDS

**DOI:** 10.1093/geroni/igae098.2827

**Published:** 2024-12-31

**Authors:** Alyssa Duckworth, Surya Venugopal, Luisa Coelho, Ester Kwon, Sameer Shah, Angels Almenar-Queralt

**Affiliations:** Sanford Consortium for Regenerative Medicine, La Jolla, California, United States; University of California San Diego, La Jolla, California, United States; University of California San Diego, La Jolla, California, United States; University of California San Diego, La Jolla, California, United States; University of California San Diego, La Jolla, California, United States; University of California San Diego, La Jolla, California, United States

## Abstract

Traumatic Brain Injury (TBI) is a serious injury that affects millions of people worldwide and has significant effects on neural pathways in the brain, making it a risk factor for neurodegenerative diseases. Most research that examines the link between TBI and neurodegeneration uses rodent models, however they do not fully recapitulate disease pathology and cannot capture human specific features including the effect of genetic variations. Human induced pluripotent stem cells (hiPSC)-derived cortical organoids have become the gold standard to model neurological disorders. They recapitulate certain aspects of physiological and neural pathways of the human brain and are easily reproducible, making them a better model. To understand the effects of TBI-induced neurodegeneration and explore potential treatment therapies, we model TBI in cortical hiPSC organoids through inducing mechanical injury. We assess AD-related morphological, biochemical, and functional downstream effects with a battery of assays. Among them, we measure the impact on neuronal activity using fluorescent Ca2+ indicators to measure Ca flux at a single-cell level post injury. Together, these assays will help us establish a human-based system to model in vitro TBI-induced neurodegeneration. We hope it can help contribute to evaluate specific therapies and diagnostic tools to stop progression of neurodegeneration after mechanical injury.

